# SsUbc2, a determinant of pathogenicity, functions as a key coordinator controlling global transcriptomic reprogramming during mating in sugarcane smut fungus

**DOI:** 10.3389/fmicb.2022.954767

**Published:** 2022-09-20

**Authors:** Shan Lu, Haoyang Zhang, Feng Guo, Yanfang Yang, Xiaorui Shen, Baoshan Chen

**Affiliations:** ^1^State Key Laboratory for Conservation and Utilization of Subtropical Agro-Bioresources, Ministry and Province Co-sponsored Collaborative Innovation Center for Sugarcane and Sugar Industry, Nanning, China; ^2^Guangxi Key Laboratory of Sugarcane Biology, College of Agriculture, Guangxi University, Nanning, China; ^3^College of Life Science and Technology, Guangxi University, Nanning, China

**Keywords:** kinase regulator, sexual mating, smut, pathogenicity, *Sporisorium scitamineum*

## Abstract

The basidiomycete fungus *Sporisorium scitamineum* is the causative agent of sugarcane smut disease. Mating between two strains of the opposite mating type is essential for filamentous growth and infection in sugarcane plants. However, the mechanisms underlying mating and pathogenicity are still not well understood. In this work we used gene disruption to investigate the role of *Ssubc2*, the gene encoding a kinase regulator in *S. scitamineum*. Deletion of *Ssubc2* did not alter the haploid cell morphology or growth rate *in vitro* or tolerance to stress, but mutants with both alleles deleted lost mating ability and infectivity. Deletion of one *Ssubc2* allele in a pair with a wild-type strain resulted in impaired mating and reduced virulence. Transcriptome profiling revealed that about a third of genes underwent reprogramming in the wild types during mating. Although gene expression reprogramming occurred in the pairing of *Ssubc2*-null mutants, their transcriptomic profile differed significantly from that of the wild types, in which 625 genes differed from those present in the wild types that seemed to be among the required genes for a successful mating. These genes include those known to regulate mating and pathogenicity, such as components of the MAPK pathway and *hgl1*. Additionally, a total of 908 genes were differentially expressed in an out-of-control manner in the mutants. We conclude that SsUbc2 functions as a key factor to coordinate the reprogramming of gene expression at the global level and is essential for the transition from monokaryotic basidial growth to dikaryotic hyphal growth through mating.

## Introduction

Sugarcane smut, first reported in Natal, South Africa, in 1877, is a fungal disease with enormous economic impact on the sugarcane industry worldwide (McMartin, 1945; Ershad and Bani-Abbassi, 1971; Leu and Teng, 1974; Akalach and Touil, 1996; Ramesh Sundar et al., 2012). A whip-like structure composed of plant tissue, fungal hyphae, and teliospores in the apex of the plant in the late stage of infection is a hallmark of sugarcane smut (Ramesh Sundar et al., 2012). *Sporisorium scitamineum*, the causative agent of sugarcane smut, has three distinct phenotypes during its life cycle: yeast-like haploid basidiospore, dikaryotic hypha, and diploid teliospore (Pombert et al., 2015). The formation of dikaryotic hyphae through the fusion of two non-pathogenic haploid basidiospores from strains of opposite mating types (MAT-1 and MAT-2) via sexual mating is required for the fungus to infect host sugarcane plants (Yan et al., 2016). Previous studies have revealed that certain genes (e.g., *Sskpp2*, *SsSln1*, *Ssprf1*, *SsAgc1*, and *SsSln1*) and cAMP-dependent protein kinase A pathways are involved in the regulation of mating/filamentation and pathogenicity (Deng et al., 2018; Chang et al., 2019; Wang et al., 2019; Zhu et al., 2019; Cai et al., 2021). However, the mechanisms underlying the regulation of mating and virulence in this fungus are still far from clear.

In the yeast *Saccharomyces cerevisiae*, the adaptor protein Ste50p, a protein kinase regulator, is necessary for pheromone-induced signal transduction and hormone-induced differentiation of cells. Ste50 bridges the downstream α-pheromone receptor (Ste2) and upstream Ste11 and Ste7 kinase cascades (Xu et al., 1996). It is also involved in regulating pseudohyphal development by regulating the kinase function of mitogen extracellular signal-regulated kinase kinase (MEKK) Ste11 (Wu et al., 1999, 2006; Grimshaw et al., 2004), a protein that also functions in hypertonicity and pheromone response (Ekiel et al., 2009; Sharmeen et al., 2019). In the human pathogen *Cryptococcus neoformans*, Ste50p is required for monokaryotic fruiting and sexual reproduction (Fu et al., 2011). In the maize smut fungus *Ustilago maydis*, a homolog of yeast Ste50, designated Ubc2, is essential for filamentous growth and virulence (Mayorga and Gold, 2001; Klosterman et al., 2008).

In this work, we used gene disruption to investigate the role of a *U. maydis* Ubc2 homolog called SsUbc2 in *S. scitamineum*. *Ssubc2* deletion mutants did not alter the morphology, growth, or response to stress in haploid basidiospores but had an adverse impact on mating and pathogenicity. Comparative transcriptome analysis revealed that SsUbc2 functioned as a master to coordinate gene reprogramming at the global level. Deletion of *Ssubc2* resulted in extensive change in gene expression patterns in monokaryotic basidiospores and in the mating process and impairment of mating, filamentation, and pathogenicity with a dose-response effect.

## Results

### Identification of *Ssubc2* in *Sporisorium scitamineum*

**FIGURE 1:**
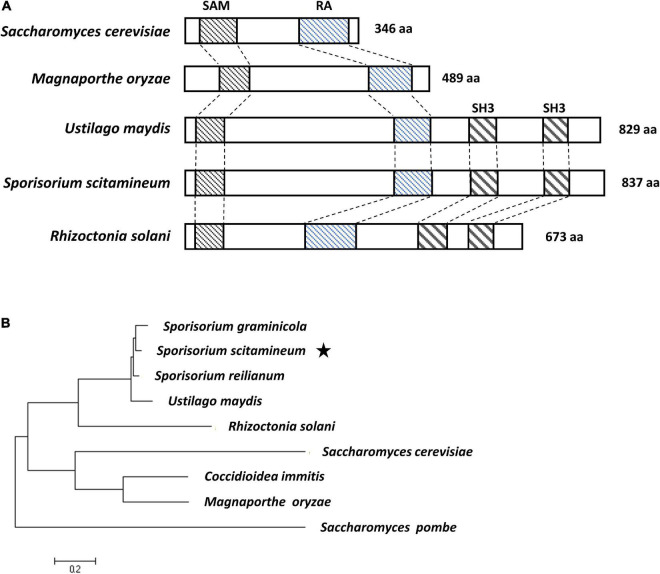
Sequence analysis of *S. scitamineum* adaptor protein SsUbc2. **(A)** Structure of the Ste50 and Ubc2 proteins. Conserved domains of Sterile Alpha Motif (SAM), Ras-Association (RA), and Src Homology 3 (SH3) are in boxes. **(B)** Phylogenetic tree constructed with amino acid sequences of Ste50 and Ubc2 proteins from ascomycetous and basidiomycetous species. The evolutionary history was inferred using the maximum likelihood method based on the JTT matrix-based model (Jones et al., 1992). Evolutionary analyses were conducted in MEGA7 (Kumar et al., 2016). Accession numbers are CBQ70036.1 for *S. reilianum*, XP_011391983.1 for *U. maydis*, XP_029739906.1 for *S. graminicola*, ELU43665 for *Rhizoctonia solani*, XP_001239789.2 for *Coccidioidea immitis*, XP_003712743.1 for *M. oryzae*, NP_009898.1 for *Saccharomyces cerevisiae*, and NP_596828.1 for *Saccharomyces pombe*. The star indicates the position of Ubc2 from *Sporisorium scitamineum*.

By blasting the nucleotide database of *S. scitamineum*^1^ using the sequence of *Ubc2* of *U. maydis* (accession no. taxid:49012) as a query, we identified a deduced protein (accession no. ON164841) with 76.3% similarity to Ubc2. The gene encoding this protein, designated *Ssubc2*, encodes a protein of 837 amino acids without any introns. Alignment with selected Ubc2 homologs from other fungi showed that SsUbc2 possesses all three domains [Sterile Alpha Motif (SAM), Ras-Association (RA), Src Homology 3 (SH3); Figure 1A]. Phylogenetic analysis revealed that Ubc2 of the basidiomycetous fungi formed a unique clade separate from Ubc2 of the ascomycetous fungi (Figure 1B).

### Phenotypic characterization and gene expression profile of *Ssubc2* deletion mutants

**FIGURE 2:**
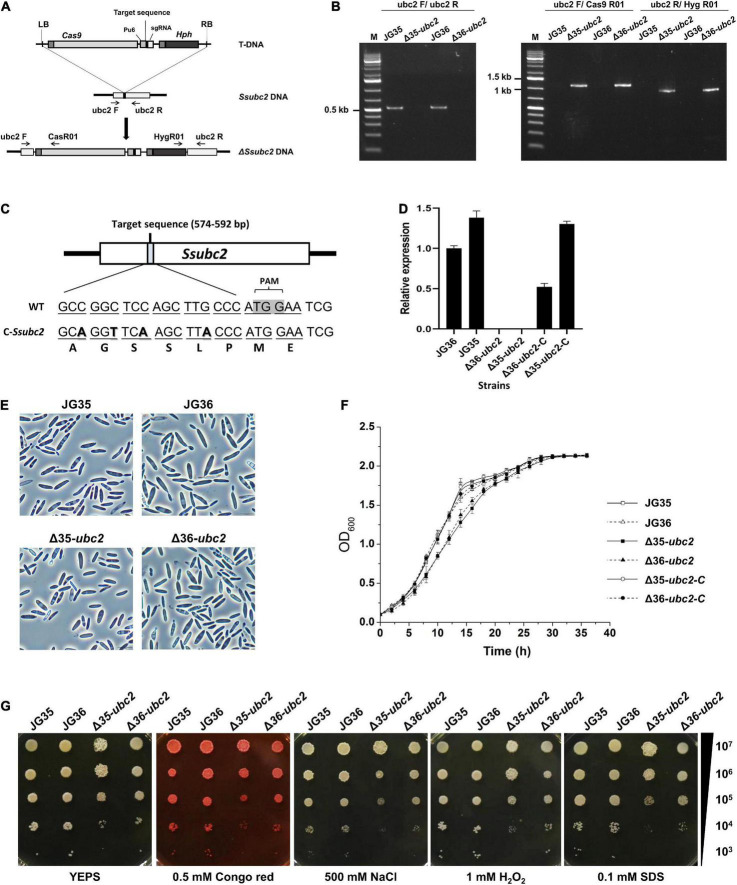
Characterization of *S. scitamineum Ssubc2* deletion and complementation strains. **(A)** Schematic representation of the *Ssubc2* gene disruption strategy. **(B)** PCR verification of insertion fragments. The primer pair ubc2F/ubc2R was used to amplify the Ss*ubc2* gene (538 bp). The primer pairs ubc2F/Cas9R01 and ubc2R/Hyg9R01 were used to amplify the left (1,087 bp) and right (878 bp) ends of the disrupted insertion fragments, respectively. **(C)** Nucleotide and amino acid sequences of wild-type and base-modified *Ssubc2* targets. **(D)** Quantification of the *Ssubc2* gene transcript in Δ*Ssubc2* mutants and complementation strains. **(E)** Microscopic images of basidiospores of wild-type strains and Δ*Ssubc2* mutants. Bar, 20 μm. **(F)** Growth rates of wild-type strains and Δ*Ssubc2* mutants. The strains were cultured in liquid YEPS medium with an initial inoculum of 1 × 10^5^ cells mL^– 1^ at 28°C with shaking at 200 rpm. Data shown are an average of three independent cultures for each strain, and error bars represent standard deviations. **(G)** Stress assays for osmolarity, ROS, and cell wall integrity. Cell concentrations are indicated at the right. Test strains were spotted onto YEPS medium supplemented with stressors and incubated at 28°C for 72 h.

Mutation of the *Ssubc2* gene in both mating types, JG36 (MAT-1) and JG35 (MAT-2), was achieved by the transformation of wild-type strains with the CRISPR-Cas9/T-DNA system for *S. scitamineum* (Lu et al., 2017; Figure 2A). We screened *Ssubc2* disruptants using PCR (Figure 2B). A total of six mutants (three for JG35 and three for JG36) were obtained. Complementation of the *Ssubc2* disruptants was achieved through the introduction of a copy of *Ssubc2* with a modified target sequence to avoid recognition by the CRISPR-Cas9 system carried by the disruptants (Figure 2C). Quantification of transcript accumulation confirmed that no *Ssubc2* expression was detected in the disruptants; *Ssubc2* expression was fully restored in the complemented Δ35-*ubc2*-C and restored up to 55% of the wild-type level in complemented Δ36-*ubc2*-C (Figure 2D). When haploid *Ssubc2*-null mutants were grown in liquid YEPS medium, no obvious defects in cell morphology, growth rate, or tolerance to stress were detected (Figures 2E–G). These results indicate that *Ssubc2* is not involved in basidial growth and may not play an essential role in hyperosmotic or oxidative stress response or in cell wall integrity.

**FIGURE 3:**
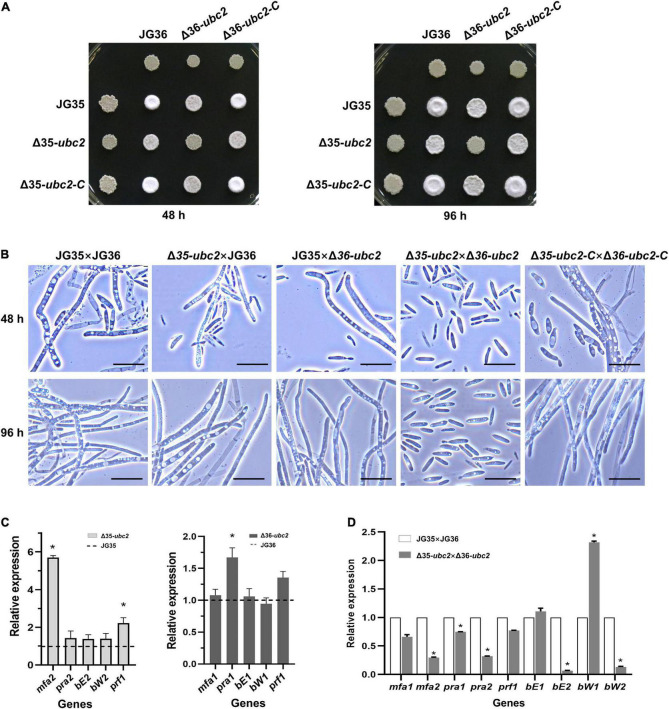
Sexual mating is attenuated in *Ssubc2* deletion mutants. **(A)** Wild-type strains, Δ*Ssubc2* mutants, and complementation strains were co-spotted on YEPS plates and incubated at 28°C for 48 h (left) or 96 h (right). Dikaryotic filaments formed colonies with a characteristic fuzzy white appearance. **(B)** Cells from a region of the colony were placed on a glass slide for observation under a microscope. Bar, 20 μm. **(C,D)** Quantification of gene transcript accumulation by quantitative real-time PCR. The relative gene expression fold change was calculated with the 2^–ΔΔCt^ method. The *actin* gene of *S. scitamineum* was used as a control. Gene expression in the wild-type haploid strains or pairs of wild-type strains was set as 1.0. *p < 0.05.

Filamentous growth is a simple indicator of successful mating between strains of smut fungi of opposite mating types. As shown in Figure 3A, co-spotting of JG35 and JG36 resulted in a fluffy white colony after 48 h of growth, whereas colonies developed from co-spotting of Δ35-*ubc2* and JG36 or of JG35 and Δ36-*ubc2* were significantly less fluffy and delayed in filamentous growth. No dikaryotic hyphae were observed from mating the two *Ssubc2* disruptants (Δ35-*ubc2* × Δ36-*ubc2*), even after 96 h (Figures 3A,B and Supplementary Table 1). However, the reintroduction of a copy of the wild-type *Ssubc2* into the Δ*Ssubc2* mutants (Δ35-*ubc2*-C and Δ36-*ubc2*-C) fully restored the mating phenotype of the Δ*Ssubc2* mutants (Figure 3A). Indeed, microscopic examination and statistical data in three independent biological replicates confirmed that *Ssubc2* deletion in both mating types prevented the mutants from mating (Figure 3B and Supplementary Table 1).

Loci *a* and *b* are known to regulate sexual mating process of *S. scitamineum*. The *mfa* and *pra* genes in the *a* locus encode pheromone and pheromone receptor that are responsible for recognition and fusion of the opposite haploid sporidia. The *b* locus harbors *bE* and *bW* genes which encode a heterodimeric transcription factor involved in dikaryotic filamentation and invasion of host plants (Zhu et al., 2019). To identify the reasons for the mating defect in the *Ssubc2* mutants, we used RT-qPCR to quantify the expression of genes in *a* and *b* loci known to be involved in the pheromone and filamentation pathway. All genes investigated were expressed at the same or higher level in the *Ssubc2* mutant strains before mating compared to the wild-type strains (Figure 3C). However, after mating, *mfa2*, *pra2*, *bE2*, and *bW2* were significantly downregulated in the mating pair Δ35-*ubc2* × Δ36-*ubc2* compared to the wild-type pair of JG35 and JG36 (Figure 3D). These results suggest that the low accumulation of pheromone, pheromone receptor, and the heterodimer transcription factor likely contributes to the lost or weak mating ability of *Ssubc2*-null mutants.

### *Ssubc2* is essential for pathogenicity

**FIGURE 4:**
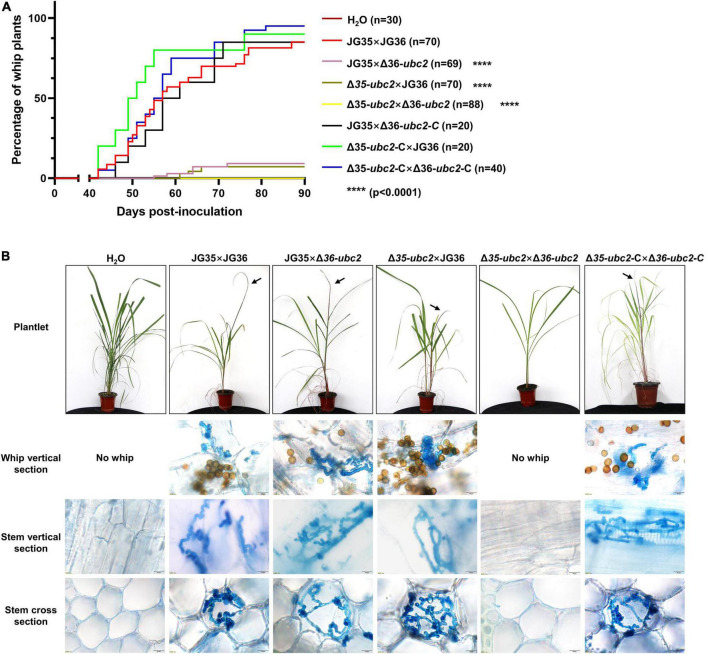
The influence of *Ssubc2* deletion on pathogenicity. **(A)** Progression of whip development induced by *Ssubc2* deletion mutants and complementation strains. Sugarcane culture tissue-derived plantlets were inoculated with combinations of wild-type strains, Δ*Ssubc2* mutants, and/or complementation strains at a concentration of OD_600_ = 1.0. Significance was set at *p* = 0.05. ****p < 0.0001. **(B)** Symptoms of plantlets inoculated with Δ*Ssubc2* mutants or wild-type strains. Arrows indicate whips. Histopathology of the plantlets was performed by dissecting the plantlets and staining them with 0.4% trypan blue. No hyphae were detected in plantlets inoculated with H_2_O or Δ35-*ubc2* × Δ36-*ubc2*. Scale bar, 20 μm.

To determine whether *Ssubc2* is required for the development of smut disease in sugarcane, we conducted virulence assays using tissue culture seedlings derived from the smut-susceptible sugarcane variety ROC22 (Lu et al., 2021). An average of 85.7% of whip development was recorded for the wild-type strain JG35 × JG36 within 90 days of inoculation, whereas only 7.1–8.7% of whip-producing seedlings were observed for Δ35-*ubc2* × JG36 and JG35 × Δ36-*ubc2* in the same period of time, and no whips were observed for Δ35-*ubc2* × Δ36-*ubc2* up to 120 days after inoculation (Figure 4A). Teliospores harvested from the whips induced by the different inoculum sources did not differ in their morphology. Histopathological examination revealed that a fraction of the inoculated plantlets was infected by Δ35-*ubc2* × JG36 (28 out of 65) or JG35 × Δ36-*ubc2* (22 out of 63), but none were infected by Δ35-*ubc2* × Δ36-*ubc2* or the control H_2_O (Figure 4B and Table 1). No obvious difference in the morphology of teliospores from whips induced by the wild-type strains or Δ35-*ubc2* × JG36 or JG35 × Δ36-*ubc2* was found. The teliospores germinated at a similar rate, with spores from Δ35-*ubc2* × JG36 and JG35 × Δ36-*ubc2* reaching 85–90% that of the wild types or the complementation strains.

**TABLE 1:** Phenotypic characterization of sugarcane plantlets inoculated with wild-type or *Ssubc2* mutant strains of *S. scitamineum*^a^.

Inoculum	No. plantlets inoculated	No. whips (rate)	No. whip-less infections	Total infection rate
JG35 × JG36	70	59 (84.2%)	1	85.7%
JG35 × Δ36-*ubc2*	69	6 (8.7%)	22	40.6%
Δ35-*ubc2* × JG36	70	5 (7.1%)	28	47.1%
Δ35-*ubc2* × Δ36-*ubc2*	88	0	0	0%
JG35 × Δ36-*ubc2-C*	20	17 (85%)	1	90%
Δ35-*ubc2-C* × JG36	20	18 (90%)	0	90%
Δ35-*ubc2-C* × Δ36-*ubc2-C*	40	38 (95%)	0	95%
H_2_O	30	0	0	0%

^a^Recorded up to 120 days after inoculation.

### *Ssubc2* influences a large number of gene expression in haploid cells

To obtain a better understanding of Ssubc2 function in haploid growth, we performed a transcriptome analysis by comparing the Δ*Ssubc2* and corresponding wild type strains under haploid condition. In total, 2,555 differentially expressed genes (DEGs) were identified in the Mat-2 strain comparison group (Δ35-*ubc2* vs. JG35) and 583 DEGs were identified in the Mat-1 strain comparison group (Δ36-*ubc2* vs. JG36), in the three biological replicates (Supplementary Figure 1). Although there was no detectable difference in haploid phenotype between the disruptants and the mutants, there were still a large number of genes whose transcription were altered, and these DEGs were mainly enriched in the cell cycle, peroxisome related pathway (peroxisomal transport, protein targeting to peroxisome, protein localization to peroxisome), membrane, catalytic activity, and G protein-coupled receptor signaling pathway (Supplementary Tables 2, 3).

### *Ssubc2* regulates gene expression at the global level and coordinates the massive change in gene expression during mating

**FIGURE 5:**
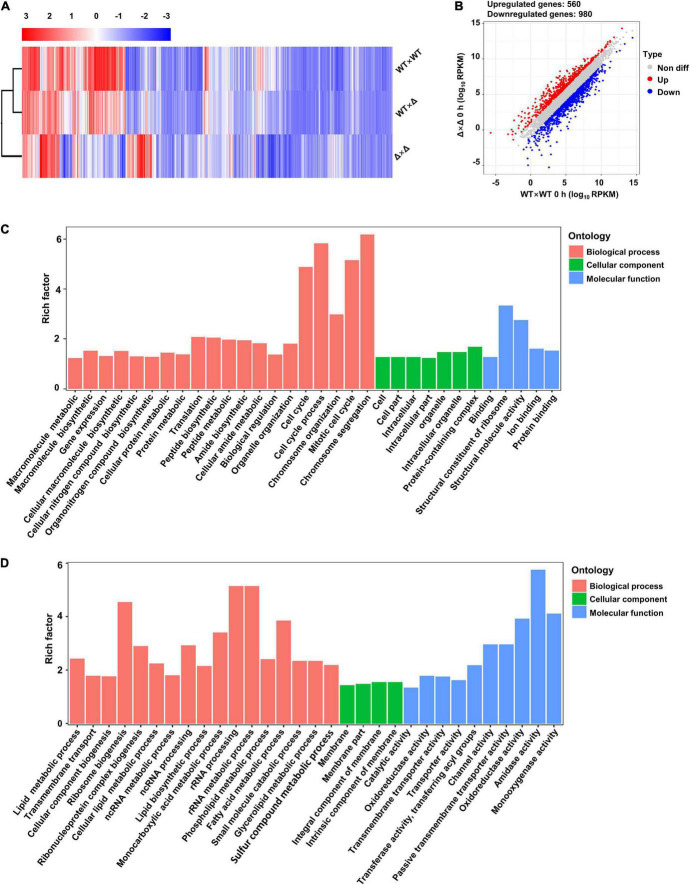
*Ssubc2* regulates the expression of a wide range of genes in the basidiospore stage. **(A)** Heat map of the RPKM values of differentially expressed genes (DEGs) of WT × WT, WT × Δ, and Δ × Δ combinations at time 0. Red indicates high expression, and blue indicates low expression. **(B)** Scatter plot of DEGs in Δ × Δ compared to WT × WT at time 0. Red dots indicate upregulated genes, and blue dots indicate downregulated genes. **(C)** Go enrichment analysis of upregulated genes in Δ × Δ compared to WT × WT at time 0. **(D)** Go enrichment analysis of upregulated genes in Δ × Δ compared to WT × WT at time 0.

To probe the mechanisms by which SsUbc2 regulates mating and pathogenicity in the fungus, we compared the transcriptomes of the wild types and the mutants in a time course manner. Profound changes in the transcription profile at time 0 were observed in the mutants compared to the wild types, with a total of 1,540 DEGs, 560 upregulated and 980 downregulated (Figures 5A,B). In terms of function, the most enriched genes were involved in cell cycle and chromosome organization in the upregulated DEGs, and the most enriched genes were involved in the rRNA and ribosome process in the downregulated DEGs (Figures 5C,D).

**FIGURE 6:**
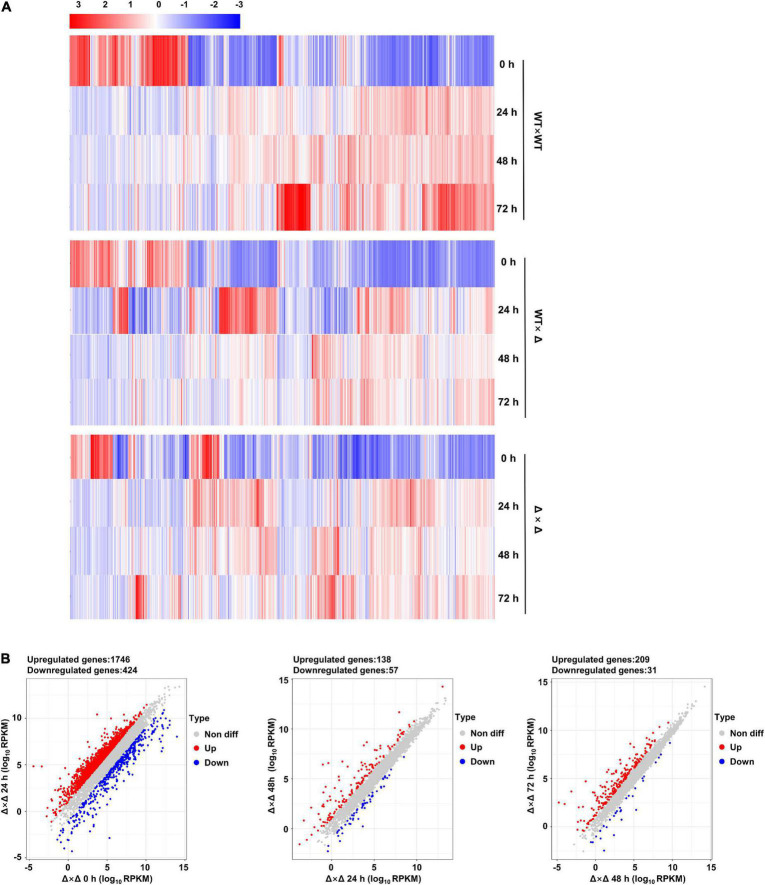
Time course of transcriptional reprogramming during mating. **(A)** Heat maps generated on the basis of RPKM values. Red indicates high expression, and blue indicates low expression. Samples were taken at 0, 24, 48, and 72 h. **(B)** Scatter plots of differentially expressed genes (DEGs) of Δ × Δ with 0 h as a reference.

Comparing transcriptomes at different time points after mating might give a complete picture of the change in gene expression. Impressive gene expression reprogramming was observed during the first 24 h of co-spoting for all three mating pairs, the wild types (WT × WT), JG35 × Δ36-*ubc2* (WT × Δ), and Δ35-*ubc2* × Δ36-*ubc2* (Δ × Δ; Figure 6A). In the case of WT × WT, 2,405 genes representing more than one third of the total genes annotated were reprogrammed compared to time 0, and much fewer DEGs were seen afterward (Supplementary Figure 1). Similar trends in gene expression reprogramming were observed in WT × Δ and Δ × Δ (Figure 6B and Supplementary Figure 2). The first sign of filamentous growth appeared 24 h after mating for the wild types, vigorous growth of the mycelium was observed at 48 h, and a steady colony was observed at 72 h. Thus, we speculate that the fungal cells experience a massive change in gene expression to respond to and cope with the mating event in the first 24 h of pairing.

**FIGURE 7:**
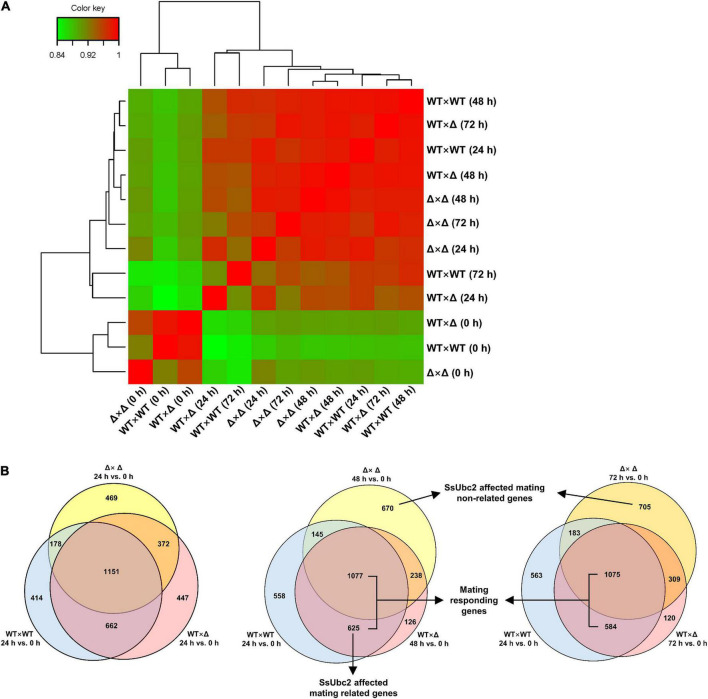
Comparison of transcriptional reprogramming during sexual mating among pairing strains. **(A)** Hierarchical cluster diagram of transcriptomes. The color scale from green to red represents lower to higher sample correlations based on gene expression. Branch lines generated by the same node indicate that the corresponding samples can be grouped into one cluster, and the length of the branch represents the similarity of the samples: the shorter the branch, the greater the similarity between samples. **(B)** Comparison of reprogrammed genes (DEGs) at different time points. DEGs were defined by reference to time 0 for each of the samples, respectively.

We then performed a transcriptome correlation analysis of WT × WT, WT × Δ, and Δ × Δ at 0, 24, 48, and 72 h. At time 0, all three pairs clustered together, but at 24 h, the wild-type pair distanced itself from the other pairs. It is intriguing that WT × Δ (48 h) and WT × Δ (72 h) clustered around WT × WT (24 h), whereas Δ × Δ mostly maintained a distance from the wild types at 24 h and onward (Figure 7A). Because 24 h after mating is considered crucial for reprogramming gene expression to make the transition from yeast-type growth to filamentous growth, we further analyzed the reprogrammed genes (DEGs) by comparing their expression with expression at time 0. Venn analysis of DEGs identified a set of 1,813 genes shared by both WT × WT and WT × Δ at 24 h, representing 68.88% of the DEGs in WT × Δ (designated as DEGs/WT × Δ/24). However, 82.38% of the DEGs/WT × Δ/48 and 79.45% of the DEGs/WT × Δ/72 were shared with the DEGs/WT × WT/24 (Figure 7B), which suggests a delay in the change in gene expression during sexual mating in WT × Δ. The shifts of DEGs in WT × Δ were in accordance with the transcriptome profiles shown in Figure 7A. In contrast, the mating-defect mutant pair Δ × Δ shared only a relatively constant portion of DEGs with WT × WT: 53.04, 50.56, and 47.32% at 24, 48, and 72 h (Figure 7B). Although only a single sample at a time point was taken for the analysis, multiple time points at the mating course unveiled a specific set of genes that were responding in WT × WT and WT × Δ, but never in Δ × Δ, suggesting that these genes are regulated by SsUbc2.

As both DEGs/WT × Δ/48 and DEGs/Δ × Δ/48 had the highest match with DEGs/WT × WT/24, we then looked into the nature of the set of 625 DEGs shared only by WT × WT and WT × Δ but not Δ × Δ (Supplementary Table 4). GO enrichment analysis showed that all 26 kinase activity–associated genes, all nine signal transduction–associated genes, and 21 out of the 32 transcription-associated genes were upregulated, whereas 11 out of the 32 transcription-associated genes were downregulated (Table 2). KEGG pathway enrichment analysis showed that all seven genes in MAPK signaling and 11 genes involved in PI3K-AK signaling, calcium signaling, AMPK signaling, and other signaling pathways were upregulated; all five genes in PPAR signaling, one gene in cAMP signaling, and one gene in the adipocytokine signaling pathway were downregulated (Table 3).

**TABLE 2:** Functional categories of DEGs that do not respond to mating in *Ssubc2*-null mutants.

	Function	Shared only by WT × WT and WT × Δ				
	
	GO term	No. of DEGs	Upregulated	Gene ID	Downregulated	Gene ID
Kinase associated	Kinase activity	16	16	g_000815, g_001439, g_001470, g_001476, g_001588, g_002441, g_003156, g_003450, g_003664, g_003976, g_004071, g_004156, g_004192, g_004306, g_005858, g_006323	0	
	GTPase activity; GTP binding	10	10	g_000645, g_001368, g_001950, g_001986, g_001993, g_002305, g_003162, g_005322, g_005401, g_006652	0	
Transcription associated	Transcription cofactor activity	4	3	g_002196, g_003047, g_001330	1	g_001238
	Cofactor binding	1	1	g_004423	0	
	Transcription corepressor activity	1	0		1	g_001238
	DNA binding transcription factor activity	4	3	g_004499, g_004877, g_006179	1	g_004566
	Zinc ion binding; RNA polymerase II transcription factor activity	12	6	g_006181, g_005758, g_000133, g_004877, g_002445, g_003128	6	g_001515, g_002926, g_000570, g_002497, g_006705, g_002297
	Transcription factor TFIID complex	1	1	g_002332	0	
	CCAAT-binding factor complex	1	0		1	g_004566
	Regulation of transcription	5	4	g_002233, g_005138, g_005226, g_005715	1	g_000657
	Regulation of transcription by RNA polymerase II	2	2	g_002196, g_003047	0	
	Transcription	1	1	g_001119	0	
Signal transduction associated	Small GTPase-mediated signal transduction	4	4	g_001950, g_002795, g_003162, g_005202	0	
	Intracellular signal transduction	2	2	g_003664, g_005963	0	
	Signal transduction	1	1	g_005322	0	
	Regulation of ARF protein signal transduction	1	1	g_005213	0	
	Phosphorelay signal transduction	1	1	g_006179	0	

**TABLE 3:** Signaling-involved DEGs that do not respond to mating in *Ssubc2*-null mutants.

Function	Shared only by WT × WT and WT × Δ				
	
KEGG term	No. of DEGs	Upregulated	Gene ID	Downregulated	Gene ID
PI3K-AKT signaling pathway	1	1	g_005988	0	
MAPK signaling pathway	7	7	g_004248, g_005322, g_004192, g_005202, g_002642, g_004306, g_001939	0	
PPAR signaling pathway	5	0		5	g_005724, g_002970, g_006649, g_004876, g_000462
cAMP signaling pathway	1	0		1	g_000462
Calcium signaling pathway	1	1	g_005963	0	
AMPK signaling pathway	1	1	g_005988	0	
Ras signaling pathway	1	1	g_005322	0	
Phosphatidylinositol signaling pathway	2	2	g_003685, g_005963	0	
Sphingolipid signaling pathway	1	1	g_005988	0	
FOX0 signaling pathway	1	1	g_003086	0	
mTOR signaling pathway	2	2	g_003665, g_002639	0	
Adipocytokine signaling pathway	1	0		1	g_002970
TGF-beta signaling pathway	1	1	g_005988	0	

## Discussion

Protein kinases play vital roles in the cell by phosphorylating target proteins to activate or suppress their cellular activity. Likewise, the regulation of kinase activity by kinase regulators is essential for keeping biological processes in the cell in order. Since the first kinase regulator in yeast, Ste50, was reported, many kinase regulators have been identified and demonstrated to function in the regulation of various aspects of growth and/or development—such as the osmolarity response, mating, filamentous growth, and pathogenicity—in fungi ranging from single-cell yeast to multicellular ascomycetes and basidiomycetes (Schamber et al., 2010; Yamamoto et al., 2010; Jung et al., 2011; Bayram et al., 2012; Gu et al., 2015). Ubc2, the homolog of Ste50 in the basidiomycetous fungus *U. maydis*, differs from Ste50 homologs in that it possesses two extra SH3 domains (Mayorga and Gold, 2001). Because SsUbc2 of the sugarcane smut fungus *S. scitamineum* is highly homologous to Ubc2 of *U. maydis* (Figure 1), it is assumed that these two proteins may have similar biochemical and biological functions in their respective fungi. Indeed, characterization of *Ssubc2*-disrupted mutants revealed that SsUbc2 is essential for mating, filamentation, and pathogenicity. Interestingly, there seems to be a dosage effect for SsUbc2, in that half dosage (one out of two alleles) resulted in weaker and delayed mating and filamentation and reduced virulence to the sugarcane plants, and the loss of both alleles totally abolished the ability of the fungus to mate or infect sugarcane plants (Figures 3, 4). However, unlike STE50 of *Saccharomyces cerevisiae*, SsUbc2 does not seem to be involved in the stress response in *S. scitamineum* (Figure 2).

The mechanisms by which STE50/Ubc2 regulates the cellular process have been investigated using molecular genetics. Through its RA domain, STE50 conducts cell signal transduction between activated G protein and STE11 and is also an essential component of three MAPK signaling pathways that control mating reaction, invasion/filament growth, and the HOG pathway, respectively (Sharmeen et al., 2019). This domain interacts with the small GTPase Cdc42 to activate the Ste11p/Ste7p/Kss1p MAP kinase cascade to control filamentous growth (Truckses et al., 2006). SH3 of Ubc2 acts as a modular component and has been implicated in mediating protein–protein interactions in receptor signaling processes, regulating enzyme activity, facilitating complex formation, and changing the subcellular localization of signaling pathway components (Bar-Sagi et al., 1993; Li, 2005; Sharmeen et al., 2019). However, one question remains: On what scale does Ubc2 influence the cellular function of a fungus?

By taking advantage of *Ssubc2*-null mutants and the half dosage mating pair (one out of two alleles) and RNA-seq technology, it could be possible to estimate the scale of the impact of SsUbc2. To our surprise, deletion of *Ssubc2* resulted in a change in expression of more than 2,000 genes, some of which are essential for the fate of the cell (e.g., cell cycle, rRNA processing and ribosome biogenesis, and nitrogen metabolism) at the basidial stage (Figure 5). Thus, we speculate that SsUbc2 may function at a top level of the kinase regulation hierarchy. Despite its extensive impact on the transcriptome, deletion of *Ssubc2* does not seem to have an apparent impact on the yeast-type growth of basidiospores (Figure 3). We further investigated the behavior of *Ssubc2*-null mutants in mating. With time 0 as a reference, the number of DEGs at 24, 48, and 72 h was 2,530, 2,130, and 2,272, respectively (Figures 6, 7B), which suggests that the mutants can detect signals from the opposite strains and respond at the transcription level accordingly. However, this transcriptional response was not in accordance with the wild-type strains or with the mating pair involving a mutant and a wild-type strain. With the DEGs of the wild types 24 h after mating as a reference, just about 50% of the DEGs of the mutants matched those of the wild types. It is interesting that about 80% of the DEGs from the pair with a half dose of SsUbc2 (WT × Δ) matched those of the wild types. Because a positive mating could be achieved in WT × Δ, we assumed that some of the genes shared by WT × WT and WT × Δ were required for mating. In this regard, the mutants failed to orchestrate some 600 genes that appear to be required for mating, although some 1,000 genes did respond (Figure 7B and Supplementary Table 4). An inspection of these 625 genes revealed many kinases, transcription factors, and signal transduction components—including hgl1 (g_003889), Rho (g_00195), Mck1 (g_004306), and Ssk2 (g_0041932)—which are required for hyphal growth, the stress response, and sporulation in other pathogenic fungi (Durrenberger et al., 2001; Bahn et al., 2007; Jeon et al., 2008; Levin, 2011).

The impairment of the MAPK pathway and kinases caused by the lack of the *Ssubc2* gene not only affects the sexual mating of mutants but also attenuates their pathogenicity to the host plant. Deletion of one *Ssubc2* allele in the pairing strains significantly reduced the virulence to sugarcane, with only 7.1–8.7% of the plantlets developing whip symptoms, even if the ability to mate was not much affected. Furthermore, when both alleles were deleted in both mating partners, the pathogenicity to sugarcane plantlets was completely abolished (Figure 4). This is distinct from the genes *Sskpp2* and *Ssprf1* and component genes of cAMP pathway, which reduce pathogenicity possibly by impairing mating/filamentation (Deng et al., 2018; Zhu et al., 2019), suggesting that the influence of *Ssubc2* deletion on pathogenicity is not entirely due to the weakened ability to form dikaryotic hyphae.

**FIGURE 8:**
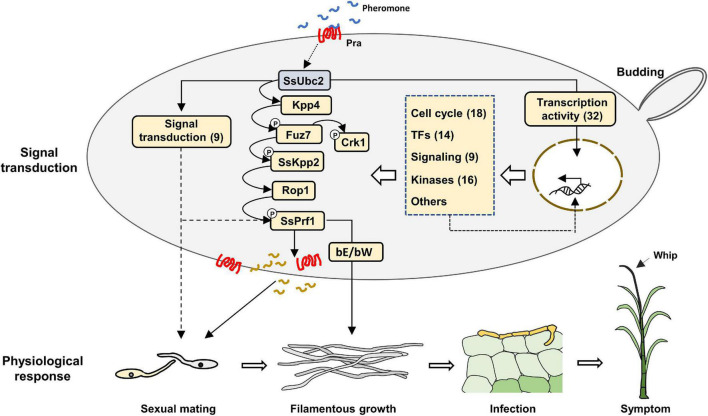
Proposed working model of SsUbc2 in *S. scitamineum*. SsUbc2 functions as a coordinator to orchestrate the transcriptome of the fungus by regulating key transcription factors that have a global impact on the transcriptome. More secondary transcription factors, protein kinases, signaling components, and other essential genes initially regulated may further exert their influence on transcription to refine gene regulation and cell function, including responses to mating and infection. Loss of SsUbc2 causes chaos in the regulation of gene expression, resulting in impairment in key signaling transduction pathways such as the MAPK pathway and dysfunction in the cell that further results in failed mating/filamentation and pathogenicity (Brefort et al., 2009). TFs, transcription factors. Arabic numerals indicate the number of DEGs.

Because Ubc2 does not seem to possess a nuclear translocation signal (Figure 1), its direct involvement in gene transcription could be largely ruled out. A more likely possibility is that high-level transcription factor(s) in the transcription regulation hierarchy is activated by a kinase with Ubc2 as an adaptor to exert its broad-spectrum effect on the fungal transcriptome. Atf1 orthologs, bZIP-type transcription factors, play an important role in vegetative growth, sexual and asexual development, the stress response, secondary metabolite production, and virulence in both human and plant fungal pathogens (Leiter et al., 2021). We noticed that many genes that encode transcription factors or transcription-related proteins and protein kinases were among the DEGs regulated by SsUbc2 upon mating (Tables 2, 3). These DEGs likely represent events downstream of the initial SsUbc2 function, but they could amplify the effect of SsUbc2. Thus, we propose that SsUbc2 functions as a coordinator to orchestrate the global transcriptome of the fungus by regulating phosphorylation of key substrate proteins in the core of the transcription regulation network. The broad and extensive impact on the transcriptome observed in *Ssubc2*-null mutants may result from the exaggeration of many downstream regulators, such as kinases and transcription factors (Figure 8).

Because *Ssubc2* is required in pathogenicity and functions at the core of the regulation of gene expression in the pathogen, it appears that this gene could be an ideal target for antifungal strategies. Of importance is that the sugarcane genome does not seem to have significant sequence homology to *Ssubc2*. Given the conserved SAM and RA domains in both ascomycetes and basidiomycetes, a broad spectrum of resistance against many plant fungal diseases caused by ascomycetous pathogens (e.g., rice blast pathogen *Magnaporthe oryzea*, cotton blight pathogen *Verticillium dahlia*, and sugarcane Pokkah boeng pathogen *Fusarium* species complex) and basidiomycetes pathogens (e.g., maize smut pathogen *U. maydis*, rice sheath blight pathogen *Rhizoctonia solani*, and sugarcane smut pathogen *S. scitamineum*) could be developed by using host-induced gene silencing (HIGS) to target *Ubc2* transcripts (Hua et al., 2018; Koch and Wassenegger, 2021). In this regard, a current report showed that HIGS was highly efficient for developing transgenic lines in rice resistant to sheath blight caused by *R. solani* (Zhao et al., 2021). In fact, recombinant microRNAs expressed by cotton plant could inhibit virulent gene expression in *V. dahlia*, and the transgenic plants showed elevated resistance to cotton blight (Zhang et al., 2016). In summary, *Ssubc2* may serve as a potentially valuable target for the control of sugarcane smut.

## Experimental procedures

### Strains, plasmids, and growth conditions

Wild-type strains JG36 (MAT-1) and JG35 (MAT-2) of the sugarcane smut fungus *S. scitamineum* are haploid basidiospores that represent opposite mating types (Zhu et al., 2019). *S. scitamineum* basidiospores were cultured in liquid YEPS medium at 28°C on a rotary shaker at 220 rpm for 1 day or were plated on solid YEPS plates for 3 days (Brachmann et al., 2001). *Escherichia coli* strain DH5α (Vazyme, Nanjing, China) was plated on Luria Agar (LA) plates or in Luria Broth (LB) at 37°C and shaken in a rotary shaker at 220 rpm. *Agrobacterium tumefaciens* strain Agl1 (Sun et al., 2014) was used for fungal transformation and was grown on LA plates at 28°C or cultured in LB liquid medium with shaking at 220 rpm.

### Generation of gene deletion and complementation mutants

The *Ssubc2* gene was deleted with the CRISPR/Cas9/T-DNA system of *S. scitamineum* as described previously (Lu et al., 2017). In brief, the target sequence (5’-ggcaagctggagccggcag-3’) of *Ssubc2* was inserted between the Pu6 promotor and the sgRNA sequence by PCR with pSgRNA-SsU6 as the template and four primers, U-F and gR-R and gRT ubc2 + and U6T ubc2–, for 30 cycles. This PCR product served as a template to amplify the sgRNA expression cassette with primer pair U-Fs-*Bam*HI/gR-R-*Hin*dIII. The sequences of primers used in this study are listed in Supplementary Table 5. The sgRNA expression cassette was cloned into the *Bam*HI and *Hin*dIII restriction sites of binary vector pLS-HCas9 to yield the disruption construct pLS-ubc2. *A. tumefaciens* strain Agl1 carrying pLS-ubc2 was used to transform *S. scitamineum* JG35 and JG36 basidiospores as described previously (Sun et al., 2014).

For complementation, the target sequence of *Ssubc2* was modified by base substitution without changing the amino acid sequence to avoid it being recognized and cleaved by Cas9 in the Δ*Ssubc2* genome. Then the modified *Ssubc2* and its promoter were amplified with wild-type *S. scitamineum* genomic DNA as the template and primer pairs ubc2-pst1-F/ubc2-com-R and ubc2-com-F/ubc2-pst1-R. The products were cloned into the *Pst*I restriction site of pLS-Ncom to yield complementation construct pUbc2-com. *A. tumefaciens* strain Agl1 carrying pUbc2-com was transformed into basidiospores of *Ssubc2* deletion strains.

### Quantification of gene expression

*S. scitamineum* cells for DNA and RNA isolation were grown on solid YEPS plates at 28°C for 3 days. DNA and RNA were extracted from fungal cells with a MiniBEST Plant Genomic DNA Extraction Kit and a MiniBEST Plant RNA Extraction Kit (TaKaRa, Beijing, China) following the protocols. A PrimeScript RT Reagent Kit was used for cDNA synthesis. Gene expression was determined with TaKaRa TB Green Premix Ex Taq II on a LightCycler^®^ 480 II. The sequences of primers used for qRT-PCR are listed in Supplementary Table 5. Relative gene expression was calculated with the 2^–ΔΔCT^ method with the *S. scitamineum* actin gene as an endogenous control.

### Phenotypic characterization

*S. scitamineum* cells for stress assay were cultured in liquid YEPS medium at 28°C on a rotary shaker at 220 rpm until they reached an OD_600_ of 1.0. Ten-fold serial dilutions were made, and 1 μL of each dilution was spotted onto YEPSA medium with or without 0.5 M NaCl, 2.0 mM H_2_O_2_, 0.5 M Congo red, or 0.1 mM SDS, respectively, and incubated at 28°C for 3 days before observation.

Haploid basidiospores of *S. scitamineum* for mating assay were grown in liquid YEPS until they reached an OD_600_ of 1.0. A volume of 0.5 μL of mixture of compatible basidiospores was co-spotted onto solid YEPS medium and incubated at 28°C. Images were taken on days 2 and 4 after cultivation (Lu et al., 2021).

### RNA isolation and sequencing

*S. scitamineum* cells were collected for RNA extraction after 0, 24, 48, and 72 h of co-spotted for mating on solid YEPS plates, respectively. Total RNA was extracted from fungal cells with TRIzol reagent, and DNA was then digested with DNase I following the manufacturer’s instructions. Construction of the cDNA library, UID (Unique Identifier) RNA sequencing, and data analysis were conducted by Wuhan SeqHealth Technology (Wuhan, China). Clean reads were aligned to reference genome sequences of *S. scitamineum* JG36 (unpublished data). The expression of each gene was calculated and normalized by corresponding reads per kilobase of transcript per million mapped reads (RPKM). DEGs between samples were selected on the basis of their fold change (| log_2_[fold change] | > 1) and *p*-value (< 0.05).

### Pathogenicity assay

The pathogenicity assay was performed with the root-dipping method as described previously (Lu et al., 2021).

### Microscopy

Tissue samples were harvested from sugarcane seedlings and stained with 0.4% trypan blue according to the protocol described previously (Lu et al., 2021). Samples were visualized with an Olympus CX33 microscope operated with CellSens Dimension software.

## Data availability statement

The datasets presented in this study can be found in online repositories. The names of the repository/repositories and accession number(s) can be found below: NCBI—BioProject ID PRJNA855015.

## Author contributions

SL, HZ, and BC conceived and designed the experiments. SL, HZ, FG, YY, and XS performed the experiments. SL and HZ analyzed the data. SL and BC wrote the manuscript. All authors contributed to the article and approved the submitted version.

## Footnotes

1
https://www.ncbi.nlm.nih.gov/assembly/GCA_900002365.1
