# Supplementary material for: SsUbc2, a determinant of pathogenicity, functions as a key coordinator controlling global transcriptomic reprogramming during mating in sugarcane smut fungus
Source: Front Microbiol. 2022 Sep 20;13:954767. doi: 10.3389/fmicb.2022.954767 (PMC9530204; doi:10.3389/fmicb.2022.954767)
Supplement: Supplementary file 1 [file Data_Sheet_1.DOCX]

Supplementary Material


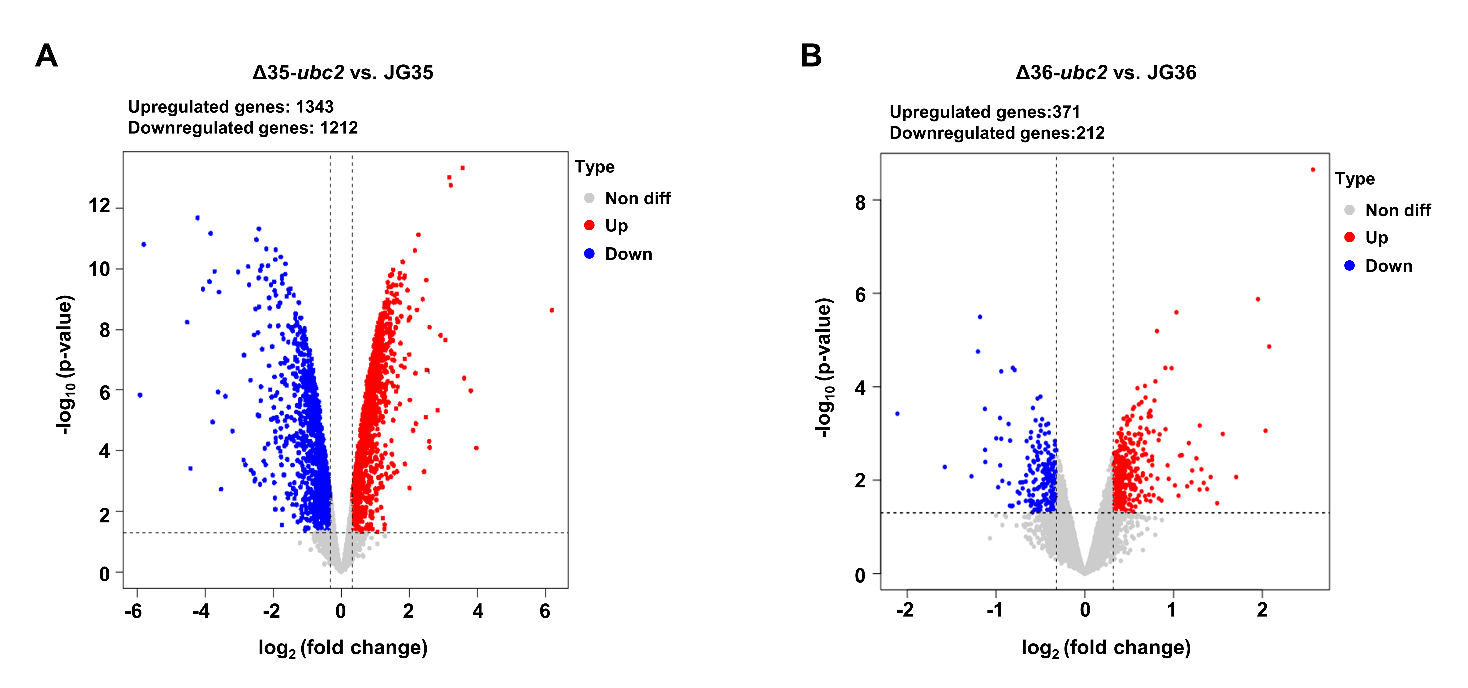
**Figure S1.** Volcano plots of DEGs of *Ssubc2* mutants and wild-type haploids. (A) Δ35-*ubc2* vs. JG35. (B) Δ36-*ubc2* vs. JG36.


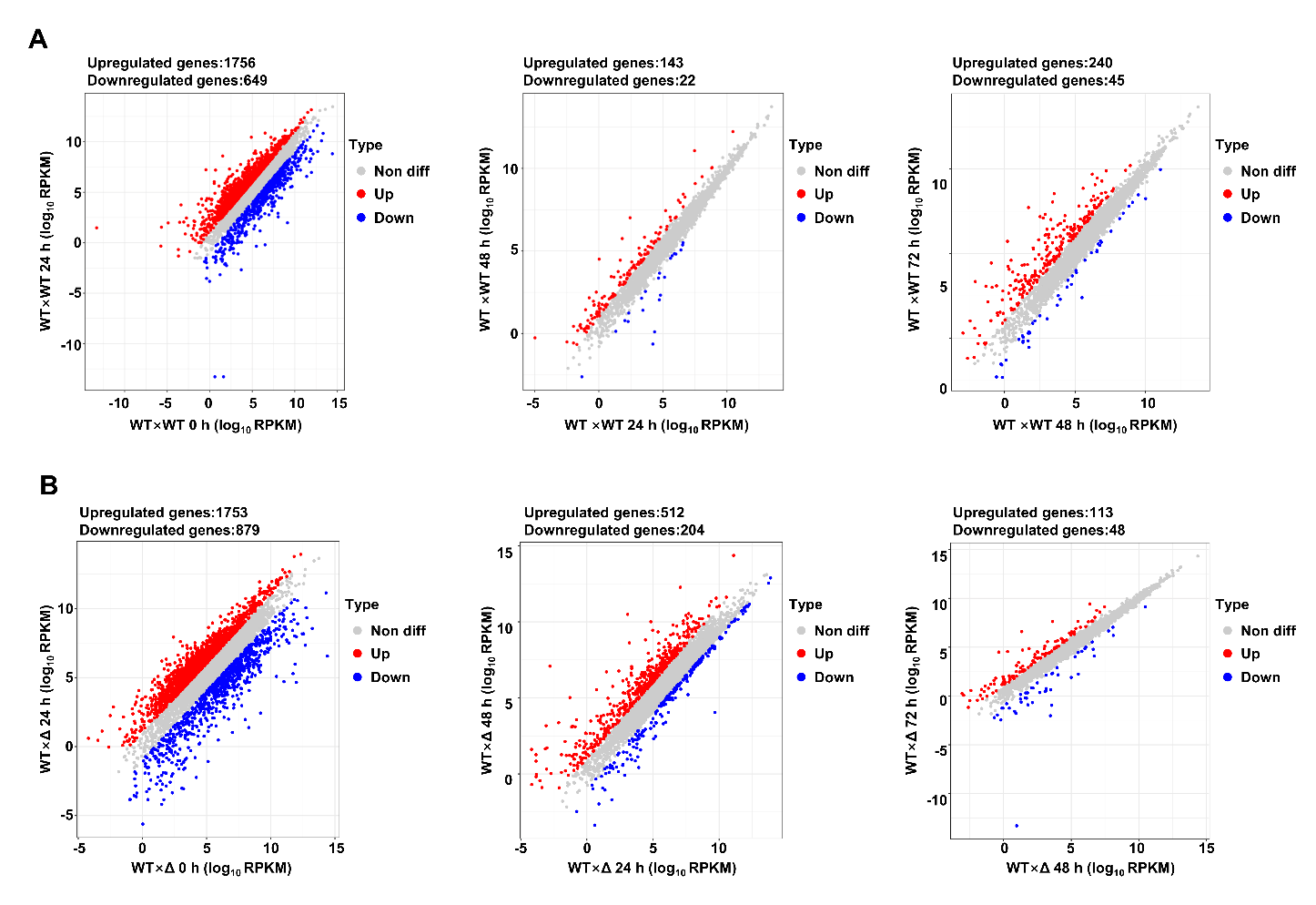
Figure S2. Scatter plots of DEGs of mutant pairs at different time points during mating. (A) WT×WT. (B) WT×Δ.
