# Supplementary material for: SsUbc2, a determinant of pathogenicity, functions as a key coordinator controlling global transcriptomic reprogramming during mating in sugarcane smut fungus
Source: Front Microbiol. 2022 Sep 20;13:954767. doi: 10.3389/fmicb.2022.954767 (PMC9530204; doi:10.3389/fmicb.2022.954767)
Supplement: Supplementary file 2 [file Table_1.DOCX]

Supplementary Material

# Table S1 The statistical data for microscopic observation of mating occurrence forming of ∆35-*ubc2*×∆36-*ubc2*

|  | No. of cells | No. of haploid | No. of hypha |
| --- | --- | --- | --- |
| ∆35-*ubc2*×∆36-*ubc2-1* | 86 | 86 | 0 |
| ∆35-*ubc2*×∆36-*ubc2-2* | 85 | 85 | 0 |
| ∆35-*ubc2*×∆36-*ubc2-3* | 84 | 84 | 0 |
| Total | 255 | 255 | 0 |
