# Supplementary material for: SsUbc2, a determinant of pathogenicity, functions as a key coordinator controlling global transcriptomic reprogramming during mating in sugarcane smut fungus
Source: Front Microbiol. 2022 Sep 20;13:954767. doi: 10.3389/fmicb.2022.954767 (PMC9530204; doi:10.3389/fmicb.2022.954767)
Supplement: Supplementary file 6 [file Table_5.DOCX]

Supplementary Material

# Table S5 List of primers used in this study

| Primer | Sequence (5′–3′) | |
| --- | --- | --- |
| U-F | ctccgttttacctgtggaatcg | Used for *Ssubc2* deletion |
| gR-R | cggaggaaaattccatccac |  |
| gRT ubc2+ | ggcaagctggagccggcaggttttagagctagaaatag |  |
| U6T ubc2- | ctgccggctccagcttgcccgagggtaaaatctgattgtatg- |  |
| gR-R | taaccatggtaccaagcttattccatccactccaagctcttg |  |
| U-Fs BamHI(in) | ctatgttactagaggatcccggaatgatctacaaagcgttcttc |  |
| Cas9 R01 | ggataccgaccttccgcttcttc |  |
| HygR01 | tgtatggagcagcagacgcgctac |  |
| ubc2-F | tccaggaaggcgattgggtg |  |
| ubc2-R | ttggaagtgctggcagcgtacg |  |
| ubc2-LA-F | aggtcaaggcgacgaaaagt | Used for *Ssubc2* complementation |
| ubc2-RA-R | accgctgttggataaccgtc |  |
| ubc2-pst1-F | gcttgcatgcctgcagtggtagacgtgccgagaat |  |
| ubc2-com-R | ccatgggtaagcttgaacctgcagtggaagcaaggtcggaattggatgc |  |
| ubc2-com-F | ttccactgcaggttcaagcttacccatggaatcgcccacttcacacaac |  |
| ubc2-pst1-R | cctctagagtcgaccatcaaatctgattgccctgccca |  |
| DL-ubc2-F | tcattcttccgagagcagcg | Used for RT-qPCR |
| DL-ubc2-R | aagttgtgtgaagtgggcga |  |
| Actin-For | cagctcgatgaaggtcaagat |  |
| Actin-Rev | cacatctgctggaaggtagag |  |
| pra1 F01 | ccacgtaacacctttctttgcgc |  |
| pra1 R01 | tcaattcgcaataacccggagc |  |
| pra2 F01 | aacgcttctttcggtgttttgagc |  |
| pra2 R01 | atcgcagccgaaagtccatgtc |  |
| mfa1 CDS F | atgctttccatctttacccagaccg |  |
| mfa1 CDS R | ttaggcgatggtgcagctagagtag |  |
| mfa2 CDS F | atgttcatcttcgagactgttgctg |  |
| mfa2 CDS R | ttaggccacggtgcagtagactgc |  |
| prf F01 | acgtcaccgtcgacctctttcac |  |
| prf R01 | ctcgcttgggaaaggagatggac |  |
| bW1 F01 | atgtcgaccactgttctatctactc |  |
| bW1 R01 | aatttgtgaaggtataggagtcg |  |
| bW2 F01 | atgtcagcctttaactcgtccatac |  |
| bW2 R01 | tacgcgtcgaggtatgcaaaactg |  |
| bE1 F01 | atggcgcaacacagtagcttcgag |  |
| bE1 R01 | cagcttgccgtgcaaagatggttgtc |  |
| bE2 F01 | gccgaccaacagcttccgattc |  |
| bE2 R01 | gcagcttgacttgcttcgaacacc |  |
